# Role of CxxC-finger protein 1 in establishing mouse oocyte epigenetic landscapes

**DOI:** 10.1093/nar/gkab107

**Published:** 2021-02-23

**Authors:** Qian-Qian Sha, Ye-Zhang Zhu, Yunlong Xiang, Jia-Li Yu, Xiao-Ying Fan, Yan-Chu Li, Yun-Wen Wu, Li Shen, Heng-Yu Fan

**Affiliations:** Fertility Preservation Laboratory, Reproductive Medicine Center, Guangdong Second Provincial General Hospital, Guangzhou 510317, China; Life Sciences Institute, Zhejiang University, Hangzhou 310058, China; Center for Stem Cell Biology and Regenerative Medicine, MOE Key Laboratory of Bioinformatics, THU-PKU Center for Life Sciences, School of Life Sciences, Tsinghua University, Beijing, China; Life Sciences Institute, Zhejiang University, Hangzhou 310058, China; Bioland Laboratory (Guangzhou Regenerative Medicine and Health GuangDong Laboratory), Guangzhou 510005, China; Fertility Preservation Laboratory, Reproductive Medicine Center, Guangdong Second Provincial General Hospital, Guangzhou 510317, China; The Second School of Clinical Medicine, Southern Medical University, Guangzhou, 510515, China; Life Sciences Institute, Zhejiang University, Hangzhou 310058, China; Life Sciences Institute, Zhejiang University, Hangzhou 310058, China; Life Sciences Institute, Zhejiang University, Hangzhou 310058, China

## Abstract

During oogenesis, oocytes gain competence and subsequently undergo meiotic maturation and prepare for embryonic development; trimethylated histone H3 on lysine-4 (H3K4me3) mediates a wide range of nuclear events during these processes. Oocyte-specific knockout of *CxxC*-finger protein 1 (CXXC1, also known as CFP1) impairs H3K4me3 accumulation and causes changes in chromatin configurations. This study investigated the changes in genomic H3K4me3 landscapes in oocytes with *Cxxc1* knockout and the effects on other epigenetic factors such as the DNA methylation, H3K27me3, H2AK119ub1 and H3K36me3. H3K4me3 is overall decreased after knocking out *Cxxc1*, including both the promoter region and the gene body. CXXC1 and MLL2, which is another histone H3 methyltransferase, have nonoverlapping roles in mediating H3K4 trimethylation during oogenesis. *Cxxc1* deletion caused a decrease in DNA methylation levels and affected H3K27me3 and H2AK119ub1 distributions, particularly at regions with high DNA methylation levels. The changes in epigenetic networks implicated by *Cxxc1* deletion were correlated with the transcriptional changes in genes in the corresponding genomic regions. This study elucidates the epigenetic changes underlying the phenotypes and molecular defects in oocytes with deleted *Cxxc1* and highlights the role of CXXC1 in orchestrating multiple factors that are involved in establishing the appropriate epigenetic states of maternal genome.

## INTRODUCTION

In mammals, female germ cells stop proliferation and enter meiosis as soon as they enter the developing ovaries in the embryos. Primordial follicles are formed when these oocytes (female germ cells in meiosis) are enclosed by a single layer of flattened ovarian pre-granulosa cells. In mouse, primordial follicles are generated at 1–3 days after birth and remain dormant for prolonged periods (1). These primordial follicles are gradually awakened during postnatal life (as early as postnatal day 5 in mouse), characterized by the dramatic increase in oocyte size, and proliferation of the granulosa cells (2). The following follicle growth and ovulation are regulated by gonadotropins secreted by the pituitary and intraovarian signaling factors (3). The female germ cells initiate meiosis shortly after entering the embryonic gonads, but they arrest at the diplotene stage of meiosis I and are enclosed by the ovarian granulosa cells. Postnatally, the oocytes in developing follicles each has a large nucleus being called germinal vesicle (GV). Therefore, they are also known as GV oocytes (4).

Oogenesis is a process during which oocytes acquire capabilities that are required for totipotency after fertilization. Mammalian oocytes are essential in preserving genetic and epigenetic information over an extended time span and transferring them to the next generation, and therefore undergo more remarkable and extensive epigenetic changes than most types of somatic cells during growth, maturation, and fertilization (5–7). Among many types of histone modifications, the trimethylation of histone H3 at lysine 4 (H3K4me3) plays a pivotal role in the epigenetic maturation of mouse oocytes and is a key determinant of the developmental potential of the resultant zygotes and early embryos (8–10). The oocyte genome not only contains narrow canonical H3K4me3 peaks at the gene promoters as in somatic cells but also establishes broad non-canonical H3K4me3 (ncH3K4me3)-deposited regions in the gene bodies. The formation of ncH3K4me3-deposited regions coincides with genome silencing (10,11).

In mammalian cells, there are six known histone methyltransferases that catalyze the trimethylation of H3K4: lysine (K) methyltransferase 2A (KMT2A), KMT2B, KMT3, KMT4, SET domain-containing 1A (SETD1A) and SETD1B (12–14). KMTs are also known as mixed lineage leukemia 1–4 (MLL1–4). The SETD1 complex does not independently bind genomic DNA; it targets chromatin through its regulatory subunit CxxC-finger protein 1, which is encoded by the *Cxxc1* gene in mammals and engages in multivalent chromatin binding to recognize both pre-existing H3K4me3 and nonmethylated DNA (15,16). Two transgenic mouse strains, *Gdf9-Cre* and *Zp3-Cre*, are most commonly used to knockout genes in mouse oocytes (17). In these two strains, Cre recombinase is expressed in oocytes as early as the primordial follicle stage and primary follicle stage, respectively (18,19). Studies using *Gdf9-Cre*-mediated *Cxxc1* knockout have revealed that SETD1-CXXC1 is one of the major KMT complexes that mediates H3K4me3 accumulation in mouse oocytes (20). Moreover, the conditional knockout of *Cxxc1* in growing oocytes using *Zp3-Cre* was reported to substantially compromise the histone exchanges, genomic DNA methylation, and transcription of the oocyte genome (21). In addition, *Cxxc1* knockout in premeiotic germ cells using a *Stra8-Cre* knock-in mouse strain (22) causes meiotic recombination defects that lead to germ cell loss and infertility in both male and female mice (23).

In addition to *Cxxc1*, oocytes deficient in *Mll2* display decreased H3K4me3 levels and abnormal maturation and gene expression, particularly of pro-apoptotic factors (24). *Mll2* knockout in oocytes results in anovulation and oocyte death. In addition, zygotic genome activation (ZGA) is compromised in the absence of *Mll2*. A recent study reported that the knockout of *Mll2* in oocytes resulted in loss of transcription-independent ncH3K4me3 but had limited effects on transcription-coupled H3K4 trimethylation or gene expression (25). However, the effects of *Cxxc1* knockout on the epigenetic landscapes of oocyte genome has not yet been investigated. Comparing the potential differences between CXXC1 and MLL2 in mediating H3K4me3 deposition on the oocyte chromatin would provide useful information for understanding the mechanisms of oocyte epigenetic maturation.

Epigenomes contain many post-translationally modified histones at different residues. Interactions between H3K4me3 and H3T3ph have been investigated biochemically and physiologically (20,26). The potential roles of H3K4 methylation in regulating other histone modifications are also under investigations (27,28). In addition to H3K4me3, H3K36 trimethylation (H3K36me3) tightly correlates with actively transcribed genome regions and also plays a role in the transcriptional activation (29). Genome-wide ChIP-seq studies show that H3K36me3 preferentially accumulates in the 3′ end of gene bodies. SET domain containing 2 (SETD2), the major methyltransferase for H3K36me3 generation, is recruited to the chromatin through the C-terminal domain of RNA polymerase II during transcription elongation (30). SETD2-dependent H3K36 trimethylation facilitates a number of processes within the cell, including splicing, repression of intragenic transcripts, and chromatin accessibility. A recent study demonstrated that SETD2 is a crucial regulator of the mouse oocyte epigenetic maturation (31). Conditional knockout of *Setd2* in oocyte leads to extensive epigenome alterations, including the failure of H3K36me3 accumulation, extension of H3K27me3 and H3K4me3 into former H3K36me3 deposited genomic regions, and abnormalities in the DNA methylome. Ultimately, maternal depletion of SETD2 results in oocyte maturation defects and subsequent one-cell arrest after fertilization (31). These phenotypes reminiscent to those observed in oocyte-specific *Cxxc1* knockout mice, and suggest a potential functional synergy of H3K4me3 and H3K36me3 during oocyte development

In this study, we performed chromatin immunoprecipitation sequencing (ChIP-seq) analyses on *Cxxc1*-null oocytes and investigated changes in the H3K4me3 landscape of oocyte genomes with deleted *Cxxc1*. Moreover, we assessed the effects of *Cxxc1*-mediated H3K4me3 deposition on other key epigenetic markers such as H3K27me3, H2AK119ub, H3K36me3 and DNA methylation in oocytes. Our results provide *in vivo* evidence that the deficiency of *Cxxc1* caused extensive epigenetic changes linked to oocyte transcriptome abnormalities and highlight the role of CXXC1 in orchestrating multiple factors involved in establishing the appropriate epigenetic states of the maternal genome during oocyte maturation.

## MATERIALS AND METHODS

### Mice

Mice of C57BL/6 genetic background were used. *Cxxc1^fl/fl^;Gdf9-Cre* mice were established by crossing *Cxxc1^fl^* allele mice with *Gdf9-Cre* transgenic mice. Both strains were previously reported (18,32). The mice were housed under specific-pathogen-free (SPF) conditions at 20–22°C, 12/12 h light/dark cycle, and 50–70% humidity; food and water were provided *ad libitum*. Experimental procedures and animal care were in accordance with the Animal Research Committee guidelines of Zhejiang University.

### Oocyte collection and culture

Twenty-one-day-old mice were injected 5 IU pregnant mare serum gonadotropin (PMSG) and euthanized 44 h later. Oocytes at the GV stage were harvested in M2 medium (M7167; Sigma-Aldrich), cultured in mini-drops of M16 medium (M7292; Sigma-Aldrich) covered with mineral oil (M5310; Sigma-Aldrich), and incubated at 37°C in a 5% CO_2_ atmosphere.

### STAR ChIP-seq library generation and sequencing

STAR ChIP-seq was used to detect H3K4me3, H3K27me3 and H3K36me3 (10). Briefly, samples were directly lysed and fragmented by incubation at 37°C for 5 min with micrococcal nuclease (MNase) and incubated with primary antibody overnight with rotation at 4°C. The next day, 100 μg Dynabeads Protein A (Thermo Fisher Scientific) was added to each sample and incubated for 2–3 h with rotation at 4°C. The beads were washed five times with 150 μl radioimmunoprecipitation assay buffer (RIPA) and once with 150 μl lithium chloride buffer. For each sample, beads were resuspended with 27 μl deionized H_2_O and 1 μl 10 × Ex-Taq buffer (Takara, RR006B); 1 μl proteinase K (catalog no. 10910000; Roche) was added to each sample, and the solutions were incubated at 55°C for 90 min to elute DNA from the beads. The samples were incubated at 80°C for 40 min to inactivate the proteinase K and were subjected to tailing, extension, ligation, and PCR library preparation without DNA purification. STAR ChIP-seq libraries and other libraries were sequenced using either the HiSeq 2500 or X Ten system (Illumina) according to the manufacturer's instructions.

### Detection of H2AK119ub1 by modified ULI-NChIP-seq

For modified ULI-NChIP-seq, 400 oocytes were used per reaction; each step was performed in triplicate. The ULI-NChIP procedure was performed as previously described (33) with the following modifications. Modified double-stranded DNA that could be digested by I-SceI were subjected to immunoprecipitation (IP). One microgram of antibody was used for each immunoprecipitation reaction. The sequence libraries were generated using NEBNext Ultra II DNA Library Prep Kit for Illumina (E7645, New England Biolabs) according to the manufacturer's instructions. The modified double-stranded DNA were digested by I-SceI after the first nine cycles of the PCR amplification. Barcoded libraries were pooled and sequenced using Illumina HiSeq X Ten platform, 150 bp paired-end reads were generated.

### Whole genome bisulfite sequencing (WGBS)

Oocytes at GV stage were collected from 3-week-old WT and *Cxxc1^fl/fl^;Gdf9-Cre* mice. Each sample was established by inoculating 200 oocytes into cell lysis buffer using mouth pipet. Then, we performed scBS-seq protocol with some modifications. WGBS libraries were then sequenced using Illumina Platform, and 150 bp paired-end reads were generated. From the oocyte samples, 27 M paired-end reads were obtained on average. Raw reads were trimmed using TrimGalore (v0.4.4) with default parameters. Subsequently, the reads were mapped against mm9 reference genome using Bismark v0.19.0 with parameters ‘–bowtie2 –non_directional’. PCR duplicates were removed. Cytosine nucleotides were distinguished as CpG, CHG, or CHH, and the methylation levels were calculated separately. We calculated the mean CpG methylation levels of various genome elements: promoter, 5′-UTR, exon, intron, 3′-UTR, gene body, intergenic, CGIs, and repeats. The methylation levels of maternal and paternal imprinting regions were analyzed by averaging the CpG methylation levels in the reported imprinting regions. The sequencing information on WGBS data used in this study are summarized in Supplementary Table S1.

### Western blot analysis

Oocytes were lysed by adding protein loading buffer and heated at 95°C for 10 min. SDS-PAGE and immunoblots were performed using a Mini-PROTEAN Tetra Cell System (Bio-Rad, Hercules, CA, USA) according to standard procedures.

### ChIP-seq data analysis

ChIP-seq reads were trimmed to 50 bp and aligned against the mouse genome build mm9 using Bowtie2 (v2.3.4.1) with default parameters. All unmapped reads, non-uniquely mapped reads, and PCR duplicates were removed. H3K4me3 peaks were analyzed using MACS2 (v2.1.1.20160309) with the parameters ‘–nomodel –nolambda –broad -B –SPMR -g mm’, and signal tracks for each sample were generated using ‘wigToBigWig’ utility of UCSC. Unique reads were then normalized by calculating the reads per kilobase (bin/peak/promoter size) per million mapped reads (RPKM). Low quality peaks (RPKM<2) were excluded. *Z*-score normalization was used to allow visualization of all samples in the same heat map. The order of bins within each group of the heat maps is random. The average intensity profiles were generated using deepTools (v2.5.4). Promoters were defined as ±2 kb regions flanking the annotated TSS. The sequencing information of ChIP-seq data used in this study are summarized in Supplementary Table S2.

### Statistical analysis

Statistical data are presented as mean ± standard error of the mean (SEM). Most experiments included at least three independent samples and were repeated at least three times. Results for two experimental groups were compared by using two-tailed unpaired Student's *t*-tests if not specifically noted in the figure legend.

## RESULTS

### CXXC1 is required for the accumulation of H3K4me3 in maternal genomic regions

**Figure 1.**
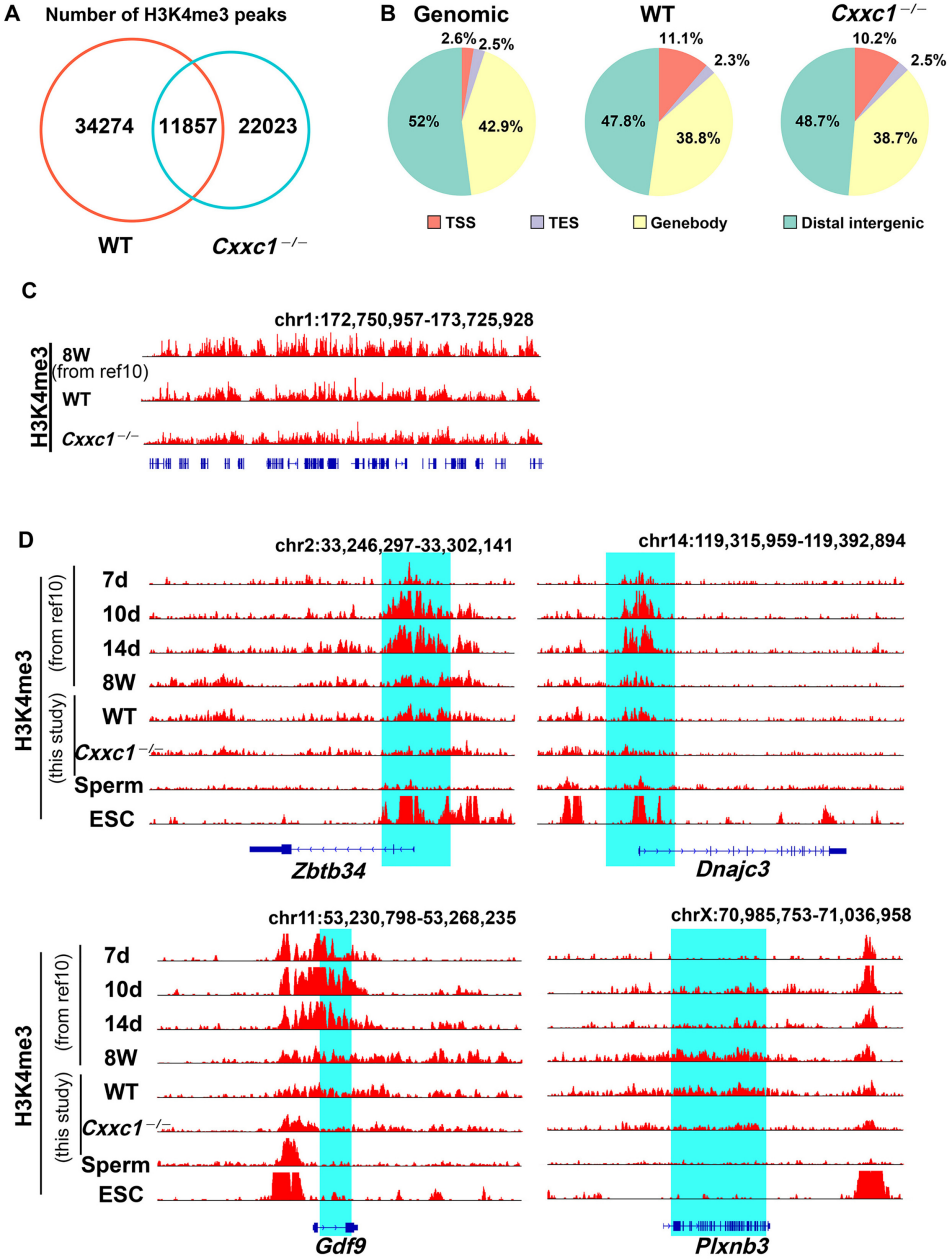
Changes in H3K4me3 states in *Cxxc1*-null oocytes. (**A**) Numbers of H3K4me3 peaks detected in wild-type (WT) (red lane) and *Cxxc1*-null oocytes (blue lane). (**B**) Genomic distribution of H3K4me3 peaks in Genomic, WT, and *Cxxc1*-null oocytes. (**C**) Snapshots from the UCSC genome browser showing the H3K4me3 enrichment in published oocytes from 8-week-old mice (10), WT oocytes in this study, and *Cxxc1*-null oocytes. (**D**) Snapshots from the UCSC genome browser showing the H3K4me3 enrichment at the indicated loci in developing oocytes (postnatal 7, 10 and 14 days), fully grown GV oocytes (8weeks) and sperms, and embryonic stem cells (ESCs) (10) fully grown GV oocytes from 4-week-old WT and *Cxxc1*-null oocytes in this study.

In previous studies, we performed the selective deletion of *Cxxc1* in postnatal oocytes by crossing *Cxxc1^fl/fl^* mice with *Gdf9-Cre* mice (*Cxxc1^oo^^–/–^*) (20,34). Immunofluorescence and western blot results indicated that H3K4me3 did not accumulate in *Cxxc1*-null oocytes of growing follicles (21,34). To investigate the changes of H3K4me3 landscape in oocyte genome with deleted *Cxxc1*, we performed ChIP-sequencing analyses using *Cxxc1*-null oocytes (100 oocytes per sample). Approximately 45 131 H3K4me3 peaks were detected in the genome of wild-type (WT) oocytes; however, among of them, only 11 857 H3K4me3 peaks were detected in *Cxxc1*-null oocytes (Figure 1A). The genomic distribution of H3K4me3 peaks located in transcription start sites (TSSs), transcription end sites (TESs), gene bodies, and intergenic regions did not change in *Cxxc1*-null oocytes (Figure 1B). As shown in the obtained snapshots from the UCSC genome browser (Figure 1C), the pattern of H3K4me3 enrichment that we detected in WT oocytes was consistent with that in published results (10), suggesting that the newly generated ChIP-seq results are reliable. The results also indicate that H3K4me3 levels were generally downregulated throughout the genome of *Cxxc1*-null oocytes, regardless of the canonical narrow peaks in the promoter regions and the noncanonical broad peaks in gene body regions (Figure 1B and C).

As determined by ChIP-sequencing analyses from previously published results (10) and this study, H3K4me3 gradually accumulated as broad peaks in the genomic regions that correspond to both the promoters and bodies of some genes at the germinal vesicle (GV) stage (Figure 1D). In the fully grown GV oocytes of 4-week-old WT mice, ChIP seq results of our own study are consistent with published results (10). in contrast, the H3K4me3 deposition in these genomic regions is either restricted to the promoter or negligible in sperms and ES cells. In oocytes isolated from *Cxxc1^oo^^–/–^* mice at GV stage, the H3K4me3 levels at these gene loci remarkably decreased, with more significantly in the promoter regions of *Zbtb34* and *Dnajc3*, or gene body regions of *Gdf9* and *Plxnb3*, respectively (Figure 1D).

### Association between histone modification in promoter regions and gene transcription changes in *Cxxc1*-null oocytes

**Figure 2.**
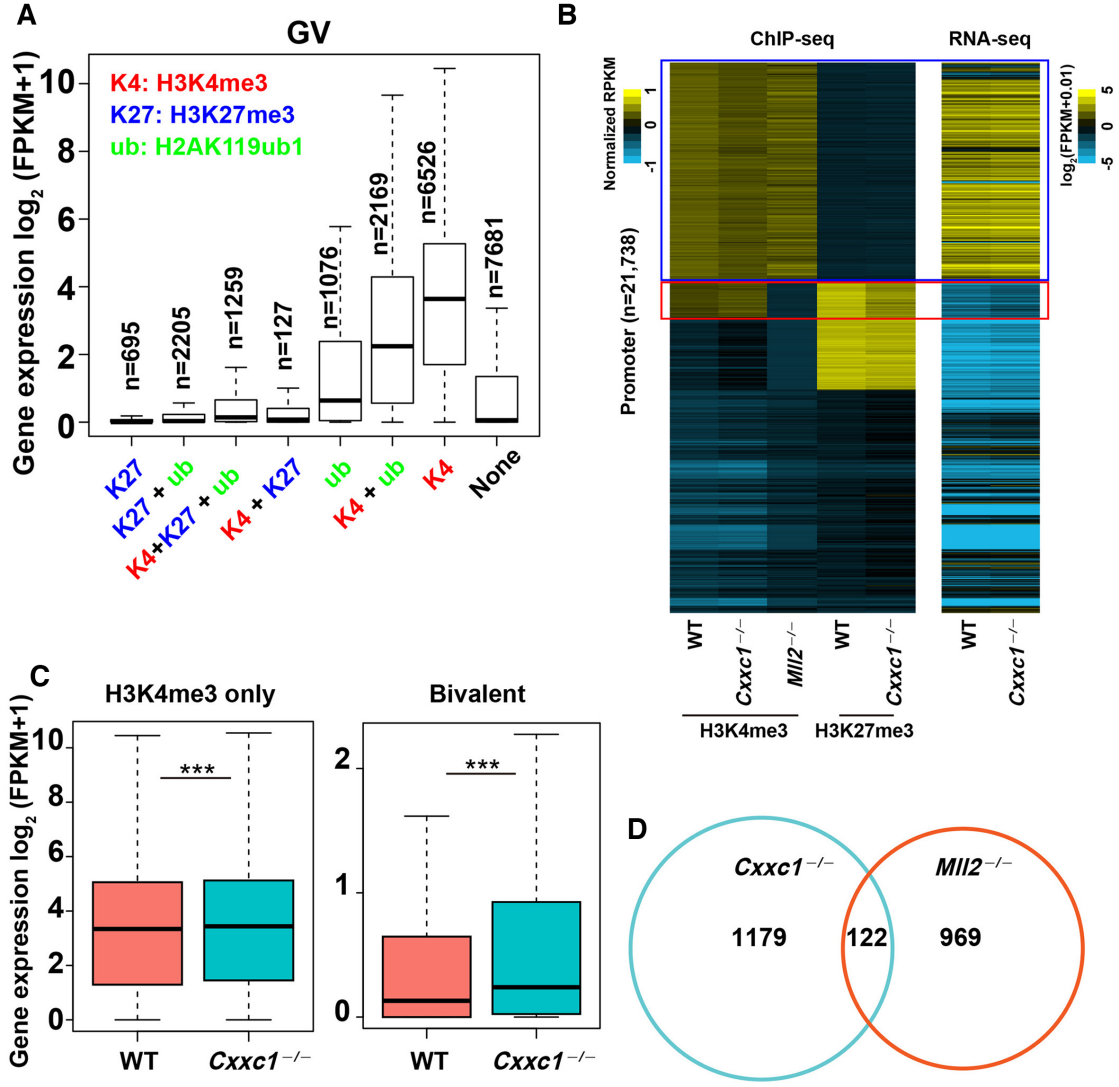
Association of epigenetic changes at the promoter and gene transcription levels in *Cxxc1*-null oocytes. (**A**) The relationship among mRNA expression levels and the enrichment of three histone modifications (H3K4me3, H3K27me3 and H2AK119ub1) at promoters in WT oocyte at GV stage. Boxplots indicate upper and lower quantiles, and the lines in the boxplots indicate the medians. (**B**) Heatmap illustrations showing the distribution of H3K4me3 and H3K27me3 at the promoter of genes and gene transcripts in oocytes according to the indicated genotypes. The promoters were grouped into four classes based on H3K4me3 and H3K27me3 levels in WT oocytes. Blue box represents H3K4me3 only regions (RPKM[H3K4me3 in WT oocyte] > 1 and RPKM[H3K27me3 in WT oocyte] < 1), and red box represents bivalent regions (RPKM[H3K4me3 in WT oocyte] > 1 and RPKM[H3K27me3 in WT oocyte] > 1). (**C**) Transcription levels of genes with H3K4me3 or H3K4me3 plus H3K27me3 accumulations in their promoter regions. Thick lines in boxes indicate the medians. The upper whisker extends from the hinge to the largest value no further than 1.5 × IQR from the hinge (where IQR is the interquartile range or distance between the first and third quartiles). The lower whisker extends from the hinge to the smallest value at most 1.5 × IQR of the hinge. ***P < 0.001 was determined using two-tailed paired Student's *t*-test. (**D**) Venn diagrams showing the overlap in the transcripts that are significantly downregulated in *Mll2* and *Cxxc1* knockout (KO) oocytes (FPKM[WT/KO] in oocytes) > 1.5).

We assessed the interrelationship of H3K4me3 with H2AK119ub1 and H3K27me3 in regulating oocyte gene expression at promoter regions (4 kb of genes). H2AK119ub1 and H3K27me3 are generated by polycomb related complexes (PRCs) (35), and were reported to maintain the silencing of a large number of genes in oocytes at GV stage. In mouse oocyte genome, 6526 promoters were exclusively enriched by H3K4me3; the genes following the promoter sequences were identified to have the highest expression levels in oocytes (Figure 2A, lane 7). The genes enriched in H2AK119ub1 or H2AK119ub1 in addition to H3K4me3 were also abundantly expressed (Figure 2A, lanes 5–6). Notably, the expression levels of genes whose gene promoters have H3K27me3 were low regardless of the presence of two other histone modifications (Figure 2A, lanes 1–4). There were 7681 genes whose promoters were not deposited with any of the three epigenetic markers. These genes have very low transcription levels (Figure 2A, lane 8). These results indicate that H3K4me3 and H3K27me3 in gene promoters strongly promotes and represses, respectively, oocyte transcription; we suggest that H2AK119ub1 has a weaker inhibitory effect than H3K27me3.

*Mll2* knockout in mouse oocytes has been reported to impair H3K4me3 accumulation and implicate oocyte development defects (24); therefore, based on published ChIP-seq results on *Mll2*-null oocytes (25) and our own results from *Cxxc1*-null oocytes, we generated enrichment profile heat maps of H3K4me3 peaks at gene promoter regions in WT, *Cxxc1* knockout, and *Mll2* knockout oocytes. Genes were sorted based on H3K4me3 and H3K27me3 levels at promoter regions in WT oocytes. The genes with high H3K4me3 but low H3K27me3 levels at their promoters were measured to show downregulation of H3K4me3 levels after *Cxxc1* deletion and were transcribed at high levels (Figure 2B, group A, boxed by blue lines). In addition to H3K4me3, *Cxxc1* knockout also resulted in decreased H3K27me3 levels at the promoters with bivalent H3K4me3 and H3K27me3 deposition (Figure 2B, group B, boxed with red lines). As shown by the RNA-seq results in the right panels of Figure 2B, the genes with H3K4me3 enrichment at their promoters were transcribed at high levels, whereas the genes with H3K27me3 enrichment at their promoters were transcribed at low levels.

Remarkably, *Mll2* knockout resulted in the converse changes in H3K4me3 levels in genes of both group A and B; H3K4me3 increased in group A, whereas H3K4me3 decreased in group B. MLL2 protein level increased in *Cxxc1*-null oocytes (34), which likely caused the deposition of additional H3K4me3 on the MLL2-targeted genome region. Meanwhile, the additional H3K4me3 might have prevented H3K27me3 accumulation at the same sites, thereby resulting in significantly increased expression of corresponding genes in *Cxxc1*-null oocytes (Figure 2B). In H3K4me3/H3K27me3 bivalent region, the decrease in H3K27me3 was associated with a significant increase in the gene expression of oocytes with deleted *Cxxc1* (Figure 2C).

In addition, the results (Figure 2B) also indicate that MLL2 is required for H3K4me3 deposition at the ‘H3K4me3/H3K27me3 bivalent and transcription-inactive promoters’, whereas CXXC1 is essential for H3K4me3 accumulation at the ‘H3K4me3-exclusive’ promoters. Furthermore, the number of downregulated genes in *Cxxc1*-null oocytes is larger than that in *Mll2*-null oocytes (1301 versus 1091), and the *Cxxc1*- and *Mll2*-regulated genes have minimal overlap in Venn diagrams (Figure 2D); therefore, we suggest that CXXC1 and MLL2 regulate different gene transcriptions.

### *Cxxc1* deletion causes DNA methylation defects in oocytes

**Figure 3.**
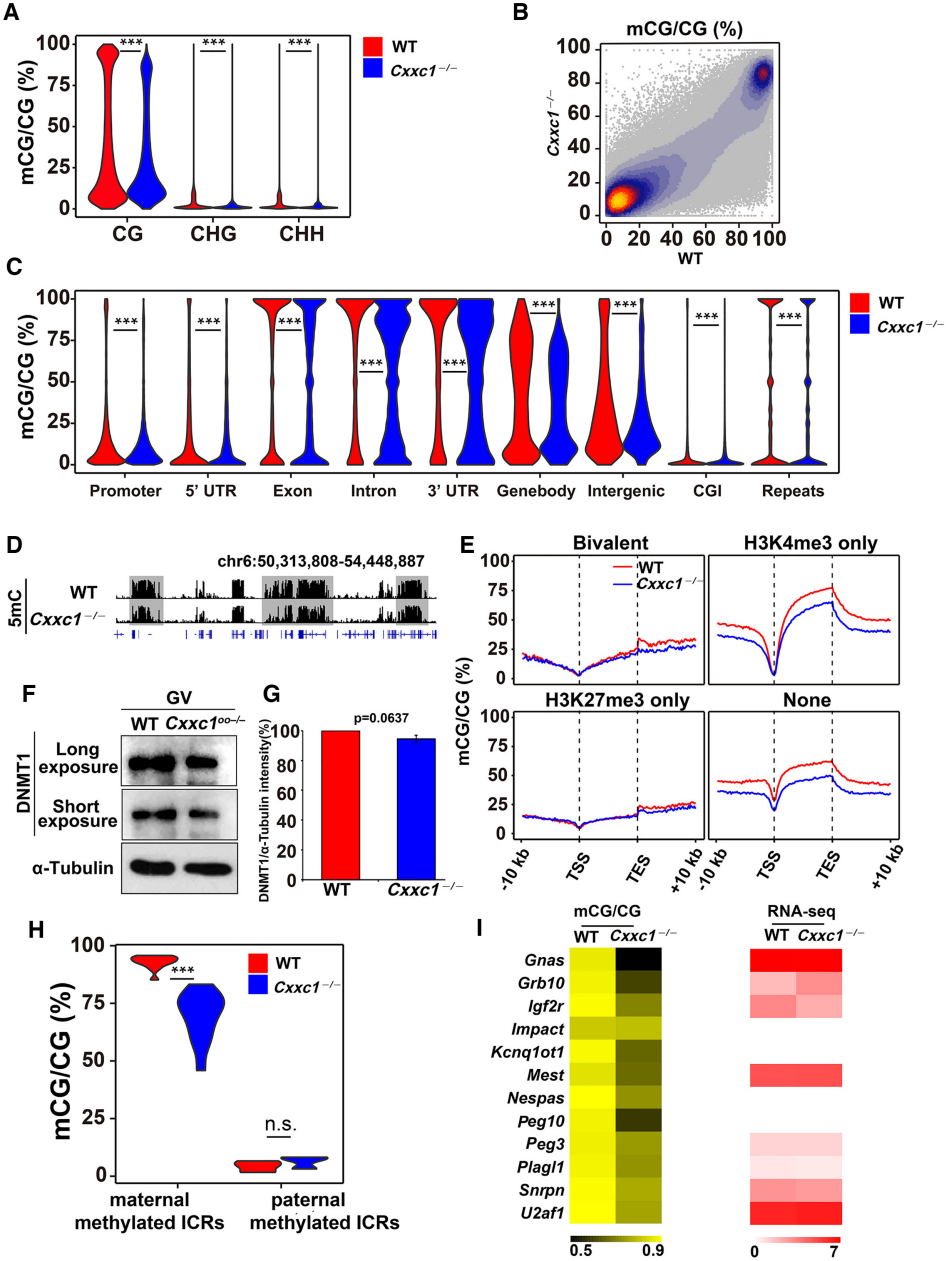
Changes in DNA methylation in *Cxxc1*-deleted oocytes. (**A**) Violin plot showing methylation levels at the CpG sites (CG), CHG, or CHH (where H correspond to A, T or C) in WT and *Cxxc1*-deleted oocytes. (**B**) Scatter plot showing methylation levels at the CpG sites in wild-type (WT) and *Cxxc1*-deleted oocytes. (**C**) Violin plot showing methylation level at different genomic regions in WT and *Cxxc1*-deleted oocytes. (**D**) Snapshots from the UCSC genome browser showing DNA methylations in WT and *Cxxc1*-null oocytes. (**E**) Enrichment profiles of DNA methylation in genes whose TSSs were deposited with the indicated histone modifications in WT oocytes. (**F**) Western blot results showing DNMT1 levels in WT and *Cxxc1*-null oocyte. α-tubulin was blotted as a loading control. (**G**) Quantification of the western blot results in (**F**) by ImageJ. (**H**) Violin plot showing the methylation levels of maternal/paternal methylated imprinting control regions in WT and *Cxxc1*-null oocytes. (**I**) Heat maps showing DNA methylation enrichment at maternal methylated imprinting control regions in WT and *Cxxc1*-null oocytes. The RNA levels for the corresponding imprinting genes are also shown. The enrichment levels are indicated by the color bars at the bottom of each pane. In (A), (C) and (H), ***P < 0.001 were determined using two-tailed paired Student's *t*-test.

During follicle growth, the oocyte genome is methylated and acquires the maternal imprint (36). CXXC1-mediated H3K4 methylation was reported to be important for maintaining DNA methylation levels in ES cells (37,38); therefore, we investigated the effect of *Cxxc1* deletion on the establishment of maternal genome imprinting. Oocytes at GV stage were collected from WT and *Cxxc1^oo^^−^^/^^−^* female mice and subjected to whole-genome bisulfite sequencing (WGBS). The C to T conversion efficiency was confirmed by using λ DNA (Supplementary Table S1). The WT and *Cxxc1*-deleted oocytes were separated by unsupervised clustering using the detected CpG methylation levels in each sample (Supplementary Figure S1A). Global DNA methylation level was decreased in the *Cxxc1*-deleted oocytes, particularly at the CpG sites that should have been highly methylated as observed in WT oocytes (Figure 3A and B). Regardless of the genomic regions, DNA methylation level was decreased in *Cxxc1*-deleted oocytes (Figure 3C). As shown in the obtained snapshot from the UCSC genome browser (Figure 3D), DNA methylation are also generally downregulated throughout the genome of *Cxxc1*-null oocytes. DNA methylation level was high at the gene body region, and the decrease in DNA methylation was significant in genomic regions without H3K27me3 in *Cxxc1*-deleted oocytes (Figure 3E); however, DNA methyltransferase 1 (DNMT1) protein was not significantly downregulated in *Cxxc1*-deleted oocytes (Figure 3F and G), suggesting that the decrease in DNA methylation was not implicated by the absence of DNMTs.

Notably, DNA methylation levels of maternally methylated imprinting control regions (ICRs) declined in the *Cxxc1*-deleted oocytes (Figure 3H). We also examined the ICRs of some important imprinting genes and confirmed the reduction of DNA methylation; however, no marked change was observed in transcription levels (Figure 3I). These results indicate that *Cxxc1* deletion in oocytes implicates the sequence-nonselective defects of *de novo* DNA methylation, especially in regions not deposited with H3K27me3. However, the partially decrease of DNA methylation levels at the maternal imprinting region may not be dramatic enough to affect gene expression.

### *Cxxc1* knockout affects H3K27me3 and H2AK119ub1 distributions in the maternal genome

**Figure 4.**
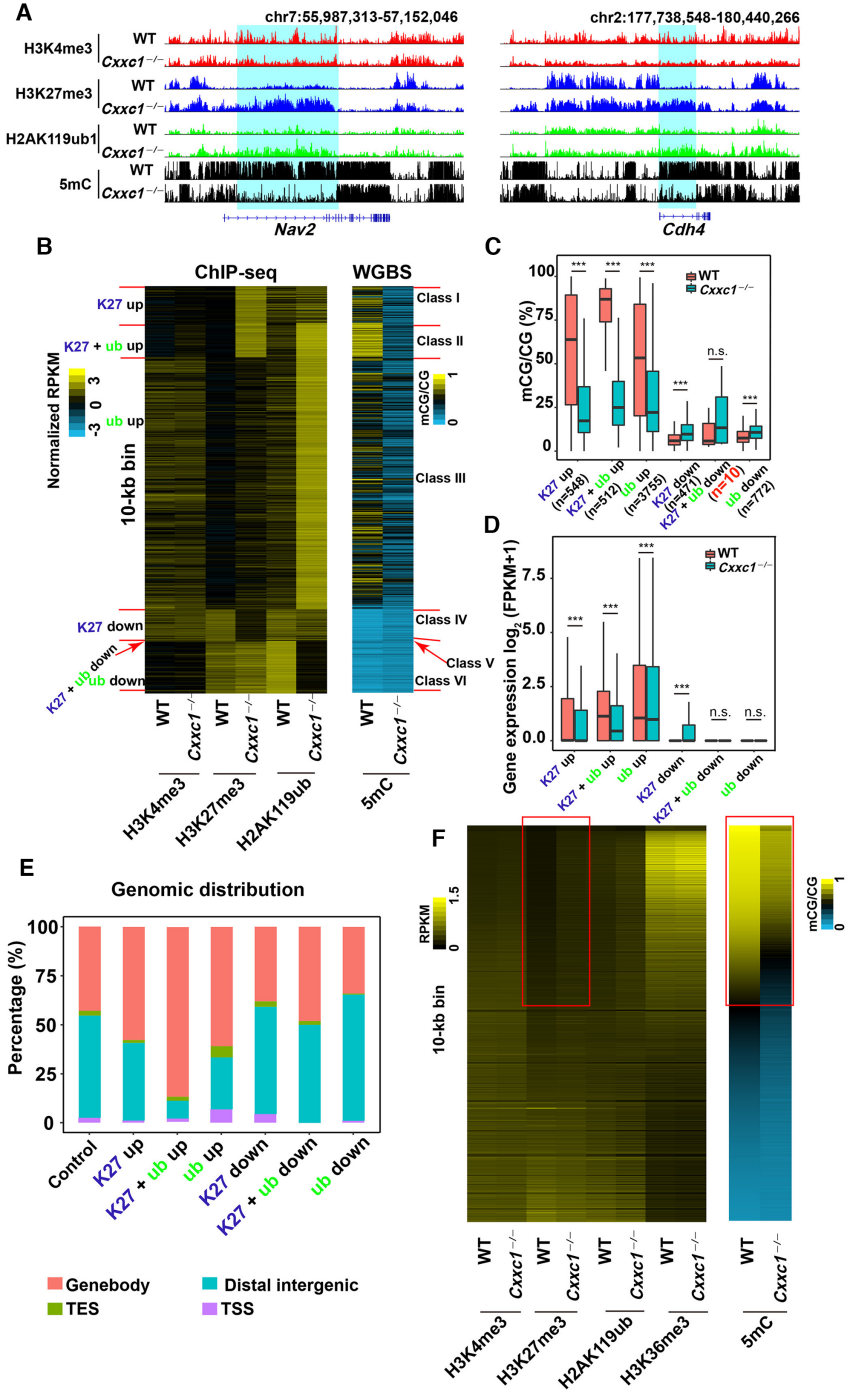
Effects of *Cxxc1* knockout on H3K27me3 and H2AK119ub1 distributions in the oocyte genome. (**A**) Snapshots from the UCSC genome browser showing the distribution of H3K4me3, H3K27me3 and H2AK119ub1 in WT and *Cxxc1*-null oocytes. (**B**) Heat maps showing H3K4me3, H3K27me3, H2AK119ub1, and DNA methylation results based on the increase and decrease of H3K27me3 and H2AK119ub1 after *Cxxc1* deletion in oocytes. Each row is a 10-kb window along the genome and only bins with changed H3K27me3 or H2AK119ub1 are shown. (**C**, **D**) The methylation levels of regions (**C**), and transcription levels of related genes (**D**) in relation to the changes of H3K27me3 and H2AK119ub1 levels after *Cxxc1* deletion in oocytes. The box indicates upper and lower quantiles, and the line in the box indicates the median. The upper whisker extends from the hinge to the largest value no further than 1.5 × IQR from the hinge (where IQR is the interquartile range or distance between the first and third quartiles). The lower whisker extends from the hinge to the smallest value at most 1.5 × IQR of the hinge. n.s., not significant, ***P < 0.001 was determined using two-tailed unpaired Student's *t*-test (C) and two-tailed paired Student's *t*-test (D). (**E**) Ratio of genomic distribution of 10-kb bins whose H3K27me3 and H2AK119ub1 levels were downregulated, upregulated, or unaffected by *Cxxc1* deletion in oocytes. (**F**) Heat maps showing H3K4me3, H3K27me3, H2AK119ub1, and DNA methylation descending ordered by DNA methylation levels in WT oocytes. Each row is a 10-kb window along the genome and only bins with at least 10% DNA methylation in WT oocytes are shown. Red box indicates high DNA methylation.

Given the mutually influences of H2AK119ub1/H3K27me3 and DNA methylation (39,40), we investigated whether the decrease in the DNA methylation levels of *Cxxc1*-null oocytes also affected the function of polycomb complexes. We analyzed the changes in H3K27me3 and H2AK119ub1 distributions throughout genomes with *Cxxc1* knockout. As shown in the snapshot of representative genomic regions wherein DNA methylation was high in WT oocytes and failed to be established after *Cxxc1* deletion (Figure 4A), H3K27me3 and H2AK119ub1 levels were low in these regions of WT oocyte genome but increased after *Cxxc1* deletion.

We also analyzed each 10-kb bin along the genome and divided the 10-kb bins showing significant changes in H3K27me3 or H2AK119ub1 levels into six major classes (Figure 4B). Class I region was not deposited with H3K27me3 in WT oocytes but acquired ectopic H3K27me3 after *Cxxc1* deletion (K27me3-up). Ectopic H2AK119ub1 deposition after *Cxxc1* deletion was acquired by class III (H2AK119ub1-up). Both ectopic H2AK119ub1 and H3K27me3 were acquired in class II (K27me3 + H2AK119ub1-up). These regions are marked by high DNA methylation levels in WT oocytes and DNA methylation deficiency after *Cxxc1* deletion. In classes IV–VI, H3K27me3, H2AK119ub1 or both were downregulated after *Cxxc1* deletion. In these regions, DNA methylation levels were low in WT oocytes but was slightly upregulated in *Cxxc1* KO oocytes (Figure 4B). We also quantitatively confirmed these results by identifying and analyzing these six classes (Figure 4C and Supplementary Figure S1B).

We further analyzed the influence of H3K27me3 and/or H2AK119ub1 changes on gene expression after *Cxxc1* deletion. When H3K27me3 and/or H2AK119ub1 levels rose, gene expressions were downregulated (Figure 4D). An increase in gene expression was observed in only the H3K27me3-down regions, whereas no change in gene expression was observed in other regions showed (Figure 4D). The increase and decrease in genomic H3K27me3 inversely correlated with the abundance of the transcripts of corresponding genes.

Shown in Figure 4E are the genomic distributions of H3K27me3 and H2AK119ub1-downregulated and -upregulated regions in *Cxxc1* KO oocytes. The majority of upregulated bins were located in gene body regions (Figure 4E, lanes 2–4). Within the gene bodies, DNA methylation also failed to proceed where H3K27me3 levels increased after *Cxxc1* deletion (Supplementary Figure S1C). H3K27me3 level was low in the DNA hypermethylated regions of WT oocytes (Figure 4F; boxed with red lines); however, in regions where DNA methylation levels were significantly decreased after *Cxxc1* deletion, H3K27me3 levels increased (Figure 4F, boxed with red lines). In the *Cxxc1*-deleted oocytes, no significant changes in H3K27me3 and H2AK119ub1 levels in the H3K27me3-related maternally imprinted genes were observed (Supplementary Figure S1D). In the *Cxxc1*-deleted oocytes, non-canonical H3K4me3, H3K27me3, and H2AK119ub1 were maintained in partially-methylated domains (Supplementary Figure S2). In summary, the distribution of H3K27me3 and H2AK119 was affected by the abnormal establishment of DNA methylation after *Cxxc1* deletion.

### *Cxxc1* knockout affects H3K36me3 distribution in the maternal genome

Histone H3K36 trimethylation (H3K36me3) is associated with transcribed regions and functions in transcription fidelity and RNA splicing (41,42). Reducing maternal H3K36me3 levels in mouse oocytes by knocking out the histone methyltransferase SETD2 results in impaired meiotic maturation and preimplantation embryo development (31). In addition, H3K36me3 participates in cross-talks with other chromatin factors; these participations include antagonizing H3K4me3 and H3K27me3 and promoting *de novo* DNA methylation by interacting with DNMTs (43,44). Furthermore, DNA methylation is lost in gene bodies upon *Setd2* knockout (31); therefore, in the following experiments, we evaluated the potential effects of *Cxxc1* knockout on the establishment of H3K36me3 landscape in mouse oocytes.

**Figure 5.**
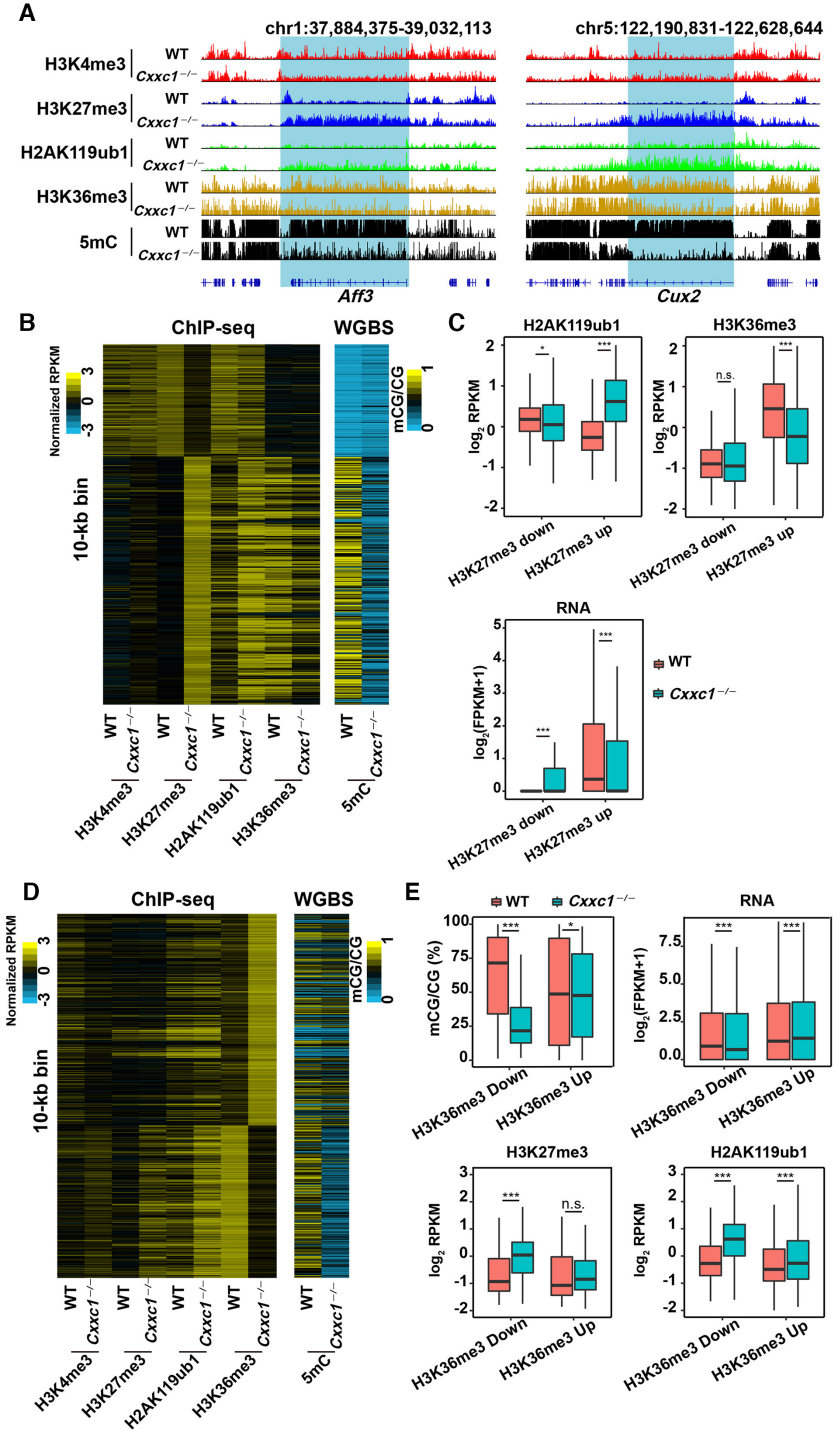
Effects of *Cxxc1* knockout on H3K36me3 distribution in the oocyte genome. (**A**) Snapshots from the UCSC genome browser showing the indicated histone and DNA methylations in WT and *Cxxc1*-null oocytes. (**B**) Heat maps showing indicated histone and DNA methylation results based on the increase and decrease of H3K27me3 after *Cxxc1* deletion in oocytes. Each row is a 10-kb window along the genome and only bins with changed H3K27me3 are shown. (**C**) Changes in H2AK119ub1 and H3K36me3 levels in H3K27me3-upregulated and -downregulated genomic regions in a 10-kb window, and RNA transcription levels of related genes after *Cxxc1* deletion in oocytes. (**D**) Heat maps showing histone modification (H3K4me3, H3K27me3, H2AK119ub1, and H3K36me3) and DNA methylation results based on the increase and decrease of H3K36me3 after *Cxxc1* deletion in oocytes. Each row is a 10-kb window along the genome and only bins with changed H3K36me3 are shown. (**E**) Changes in DNA methylation, H3K27me3, and H2AK119ub1 levels in H3K36me3-upregulated and -downregulated genomic regions in a 10-kb window and RNA transcription levels of related genes after *Cxxc1* deletion in oocytes. In (C) and (E), thick lines in boxes indicate the medians. The upper whisker extends from the hinge to the largest value no further than 1.5 × IQR from the hinge (where IQR is the interquartile range or distance between the first and third quartiles). The lower whisker extends from the hinge to the smallest value at most 1.5 × IQR of the hinge. n.s., not significant, *P < 0.05 and ***P < 0.001 were determined using two-tailed unpaired Student's *t*-test (H3K27me3, H2AK119ub1, H3K36me3 and DNA methylation) and two-tailed paired Student's *t*-test (RNA transcription).

As shown in the snapshot of representative genomic regions (Figure 5A) and heat maps showing 10-kb bins along the genome with significant changes in H3K27me3 levels (Figure 5B), after *Cxxc1* deletion, H3K36me3 levels decreased in the genomic regions where H3K27me3/H2AK119ub1 levels were upregulated and DNA methylation downregulated. We analyzed the changes in DNA methylation (Supplementary Figure S1B), H3K36me3, H2AK119ub1, and transcribed RNA in relation to H3K27me3 changes after *Cxxc1* knockout (Figure 5C). While levels of DNA methylation, H3K36me3, and RNA transcription significantly decreased in H3K27me3-upregulated bins, H2AK119ub1 levels in these bins increased. In this aspect, decreasing of H3K36me3 and DNA methylation may jointly lead to ectopic H3K27me3 and H2AK119ub1 accumulation. To analyze whether changes in the distribution of DNA methylation were due to the changes in H3K36me3 after *Cxxc1* deletion, we analyzed the changes of H3K36me3 distributions throughout 10-kb bins along the genome after *Cxxc1* knockout. Heat map showing genomic bins with significantly changed H3K36me3 levels (Figure 5D). Quantitative analysis indicated that DNA methylation was downregulated regardless of whether H3K36me3 was upregulated or downregulated (Figure 5E). In contrast, H3K27me3 and H2AK119ub1 were upregulated regardless of whether H3K36me3 was upregulated or downregulated (Figure 5E).

**Figure 6.**
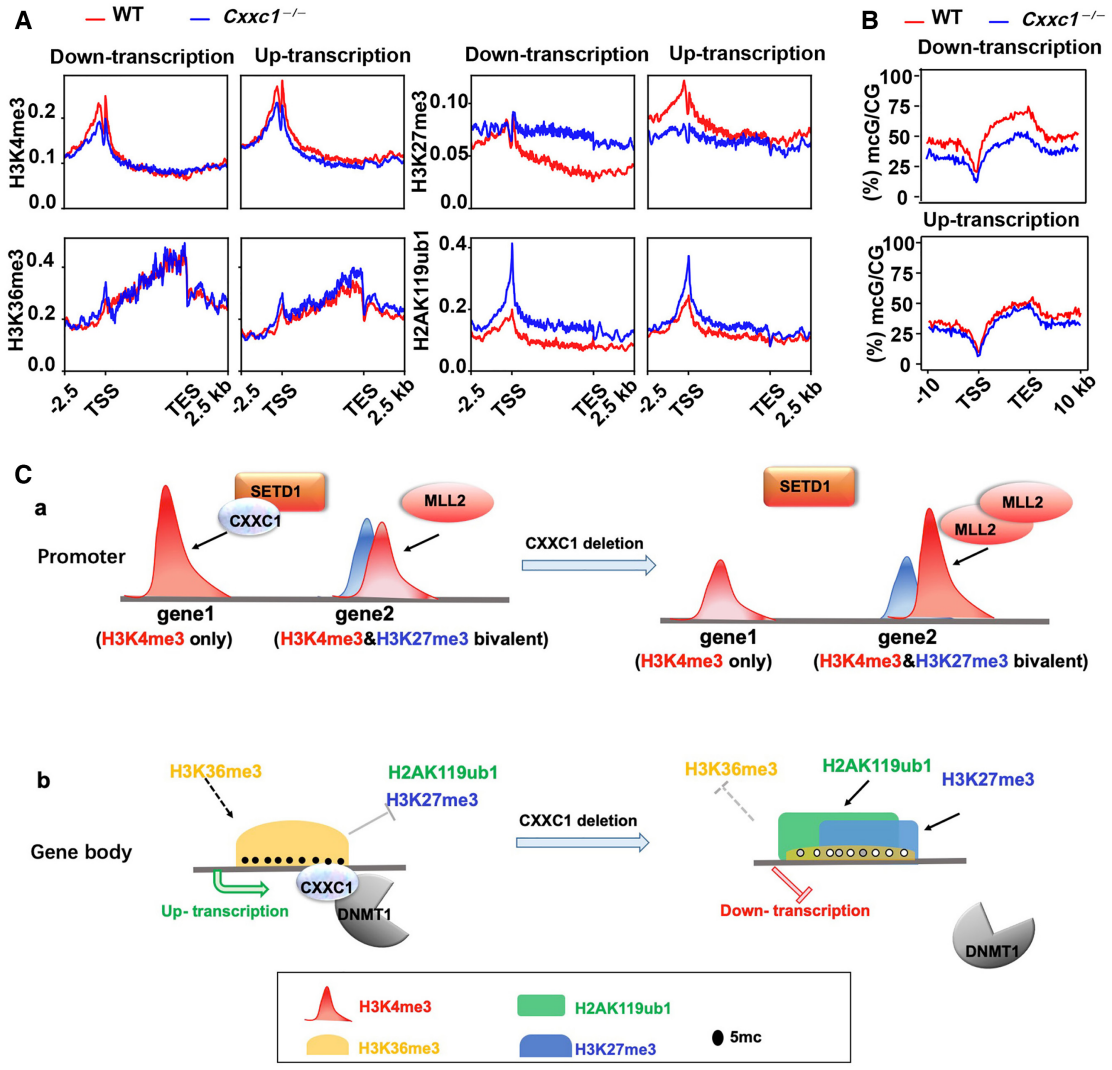
Summary of epigenetic changes in relation to transcriptional changes in normal and *Cxxc1* knockout oocytes. (A, B) Enrichment profiles of indicated histone modifications (H3K4me3, H3K27me3, H2AK119ub1 and H3K36me3) (**A**) and DNA methylation (**B**) in genes whose mRNA levels were downregulated or upregulated after *Cxxc1* knockout in oocytes at GV stage. (**C**) Model illustrating epigenetic reprogramming in oocytes regulated by CXXC1. a: CXXC1 and MLL2 have nonoverlapping roles in mediating H3K4 trimethylation at the promoter. MLL2 is required for H3K4me3 deposition at the ‘H3K4me3/H3K27me3 bivalent and transcription-inactive promoters’, whereas CXXC1 is essential for H3K4me3 accumulation at the ‘H3K4me3-exclusive’ promoters. b: *Cxxc1* deletion causes a significant decrease in DNA methylation levels in oocytes and affected H3K27me3 and H2AK119ub1 distributions at regions with high DNA methylation levels. *Cxxc1* deletion also leads to a decrease in H3K36me3 at these regions.

Finally, we summarized the changes in four epigenetic markers investigated in this study, in relation to the upregulation and downregulation of the transcriptional activities of the corresponding genomic regions in oocytes with *Cxxc1* deletion (Figure 6A). In *Cxxc1*-null oocytes regardless of whether the transcription was downregulated or upregulated, H3K4me3 was decreased at both TSS and gene bodies; H3K27me3 levels increased significantly in the gene bodies of downregulated genes but decreased at the TSS of upregulated genes; H2AK119ub1 increased more in downregulated than in upregulated genes. In *Cxxc1*-null oocytes, H3K36me3 levels did not show correlational changes with the transcriptional activities of the corresponding genomic regions (Figure 6A). There was a significant decrease in DNA methylation in the gene bodies of downregulated genes than in the upregulated genes (Figure 6B). These results indicate that the transcriptome changes in *Cxxc1*-null oocytes partially correlate with the ectopic deposition of H3K27me3 and DNA methylation.

## DISCUSSION

A previous study has shown that H3K4me3 is broadly distributed in a non-canonical form at regions such as promoters, gene bodies, and intergenic regions in fully grown mouse oocyte (8–10). In another study, the ablation of *Mll2* resulted in global loss of non-canonical H3K4me3 (25). To determine whether the pattern of H3K4me3 is altered in *Cxxc1* deficient oocyte, we performed H3K4me3 ChIP-seq in fully grown GV oocytes of WT and *Cxxc1^fl/fl^;Gdf9-Cre* mice, and compared our results with those in previous studies. Our ChIP-seq data suggests that the enrichment of H3K4me3 is overall decreased after abolishing *Cxxc1*, including both the promoter region and the gene body. The decrease in canonical promoter deposition of H3K4me3 may be the direct reason for the decreased expression of oocyte genes with *Cxxc1* knockout. *Cxxc1* knockout may also affect nucleosome occupancy in oocytes; this issue should be clarified in future investigations.

The ChIP-seq data were also consistent with our observations that H3K4me3 level decreased in oocytes and did not completely disappear after either *Mll2* or *Cxxc1* knockout, as detected using immunofluorescence and western blot (20,24). Collectively these results indicate that both CXXC1 and MLL2 are required for establishing the H3K4me3 landscape during oocyte development but have partially nonoverlapping targeting chromatin regions. For example, CXXC1 preferentially mediates H3K4 trimethylation at the gene promoters that are not occupied by H3K27me3 and facilitates the transcription of these genes. In contrast, MLL2 is mainly responsible for H3K4me3 deposition in the noncanonical genomic regions that are also occupied by H3K27me3 (Figure 6C, panel a). When *Cxxc1* was deleted in mouse oocytes, MLL2 was upregulated in the *Cxxc1* KO oocytes, presumably due to genetic dosage compensation (34). As a result, the overexpressed MLL2 could mediate the formation of extra H3K4me3 peaks in genomic regions that are normally regulated by MLL2 (Figure 6C, panel a). This hypothesis is supported by the observation that the H3K4me3 levels in these regions were downregulated in *Mll2* null oocytes (Figure 2B). MLL2-dependent H3K4 trimethylation does not affect gene expression as the targeted genes are already at a transcription-repressed state.

One of the most significant epigenetic changes implicated by *Cxxc1* knockout in oocytes is the decrease in DNA methylation levels at the CpG sites that are originally hypermethylated in WT oocytes. Although the methylation levels in maternal imprinting control regions were decreased by *Cxxc1* deletion (Figure 3H), the expression levels of those corresponding genes did not change (Figure 3I). As shown in Figure 3H, the DNA methylation levels of these maternal-imprinting genes decreased to some extent but were not completely abolished. Therefore, the effect may not be dramatic enough to affect gene expression. ChIP-seq results indicated that these regions are not abundantly deposited with H3K4me3, and the H3K4me3 levels in these regions are not remarkably affected after *Cxxc1* deletion. Furthermore, western blot results indicated that the expression level of DNMT1 protein was not affected in *Cxxc1* knockout oocytes. Therefore, the decrease in DNA methylation is not a secondary effect caused by H3K4me3 deposition changes or DNMT1 expression changes after *Cxxc1* knockout. A previous study has shown that CXXC1 directly interacts with DNMT1 and regulates DNA methylation in ES cells (38). Our results provide *in vivo* evidence that a similar mechanism may also be important for the establishment of maternal DNA methylation imprinting during oogenesis.

The WGBS method does not allow us to distinguish between 5mC and 5hmC in the genomic DNA. Therefore, 5hmC levels may also be decreased in *Cxxc1* null oocytes. However, previous studies have shown that genomic DNA has high 5mC levels and only undergoes extensive 5mC-to-5hmC transition after fertilization (45). TET3, the major enzyme that mediates 5mC-to-5hmC transition in oocytes and zygotes, is localized in the ooplasm of fully grown oocytes (Supplementary Figure S5 of (46)). The WGBS results in oocytes may mainly reflect the changes in 5mC levels.

In WT oocytes, DNA methylation sites, particularly those located in gene bodies, have an important function of maintaining gene transcription by repressing the activity of polycomb complexes and preventing the deposition of H3K27me3 and H2AK119ub1 (39,40). When CXXC1-dependent DNA methylation is absent, the H3K27me3- and H2AK119ub1-deposited regions are extended in the genome, and the transcription of genes in these regions are inhibited. Previous studies have shown that DNA methylation represses local H3K27 accumulation (39,47), but whether H3K27me3 could in turn represses local DNA methylation has not been reported. Therefore, we hypothesized that *Cxxc1* deletion causes a significant decrease in DNA methylation levels in oocytes and affects H3K27me3 and H2AK119ub1 distributions in regions with high DNA methylation levels (Figure 6C, panel b). Therefore, CXXC1 facilitates gene transcription in developing oocytes not only by mediating canonical H3K4me3 deposition at promoters but also by affecting DNA methylation and establishing other histone modifications.

In addition to the effects of *Cxxc1* knockout on H3K27me3 and H2AK119ub1 deposition in the oocyte genome, it also leads to a decrease in H3K36me3 at some genomic regions; whether this is caused by the changes in DNA methylation or PRC1/2 targets remains unclear. It is possible that the increase in H3K27me3 represses H3K36me3 deposition in the adjacent area. The transcription repression and chromatin condensation caused by H3K27me3 accumulation may prevent access of SETD2 to the local chromatin (Figure 6C, panel b). Further investigations are required to elucidate the extended epigenetic networks regulated by CXXC1 and H3K4me3 during oocyte maturation.

Based on these RNA-seq and ChIP-seq results, we analyzed the effects of single and combined histone modifications in promoters or gene bodies on gene transcriptions. Generally, H3K4me3 in promoter regions facilitates gene expression, whereas PRC1/2 targets H3K27me3 and H2AK119ub1 and represses gene expression; H3K27me3 has a stronger inhibitory effect than H2AK119ub1 in both promoters and gene bodies. As a result, H3K27me3 levels inversely correlate with gene transcription levels more than H2AK119ub1 levels.

Altogether, this study detected the changes in genomic H3K4me3 landscapes in oocytes with *Cxxc1* knockout and the effects of H3K4me3 changes on other epigenetic factors including H3K27me3, H2AK119ub1, H3K36me3 and DNA methylations (Figure 6C). Furthermore, the results indicated that CXXC1 and MLL2 have nonoverlapping roles in mediating H3K4 trimethylation at different genomic regions during oogenesis, elucidating why both histone H3 methyltransferases are required in oocyte maturation (Figure 6C). In addition, the mechanisms underlying the decrease in key gene expression events in *Cxxc1* deleted oocytes were determined according to the changes in epigenetic networks.

## DATA AVAILABILITY

WGBS and ChIP-seq data have been deposited in the NCBI Gene Expression Omnibus database under the accession code GSE159581.

## Supplementary Material

gkab107_Supplemental_File
