# Supplementary material for: Role of CxxC-finger protein 1 in establishing mouse oocyte epigenetic landscapes
Source: Nucleic Acids Res. 2021 Feb 23;49(5):2569–82. doi: 10.1093/nar/gkab107 (PMC7969028; doi:10.1093/nar/gkab107)
Supplement: gkab107_Supplemental_File [file gkab107_supplemental_file.docx]

**Supplementary Information**

**
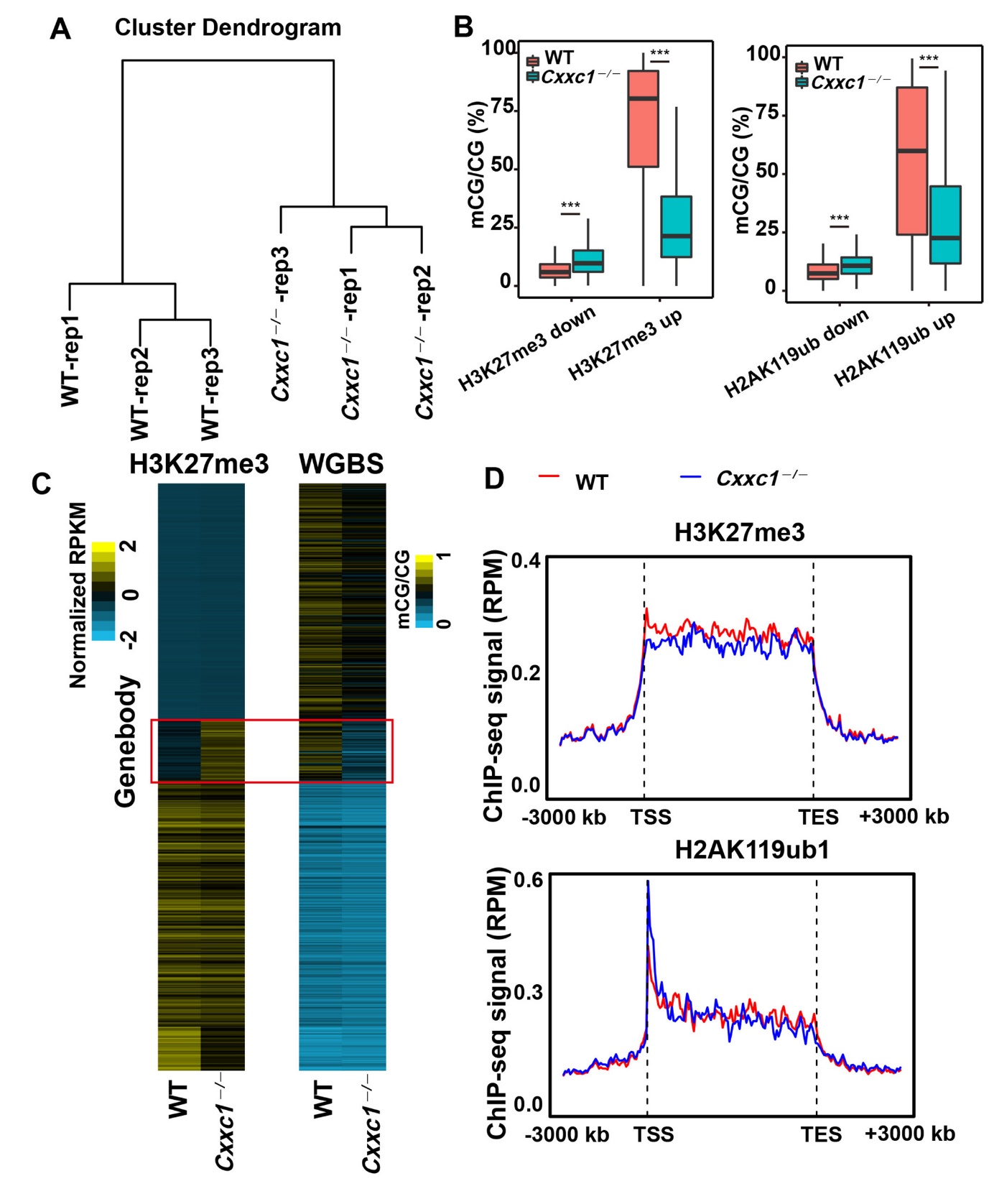
**

**Figure S1. Epigenome analyses of wild-type and *Cxxc1*-null oocytes. A:** Clustering of bisulfate-sequencing results separated wild-type (WT) and *Cxxc1*-deleted samples. **B:** Methylation levels in regions related to the changes in H3K27me3 or H2AK119ub1 levels after *Cxxc1* deletion in oocytes. The box indicates upper and lower quantiles, and the line in the box indicates the median. The upper whisker extends from the hinge to the largest value no further than 1.5 * IQR from the hinge (where IQR is the inter-quartile range, or distance between the first and third quartiles). The lower whisker extends from the hinge to the smallest value at most 1.5 × IQR of the hinge. ****P* < 0.001. **C:** Heat maps showing the indicated H3K27me3 and DNA methylation levels of gene body in WT and *Cxxc1*-deleted oocytes based on the increase and decrease of H3K27me3. **D:** Enrichment profiles of H3K27me3 and H2AK119ub1 in H3K27me3-dependent imprinting genes in WT and *Cxxc1*-null oocytes.


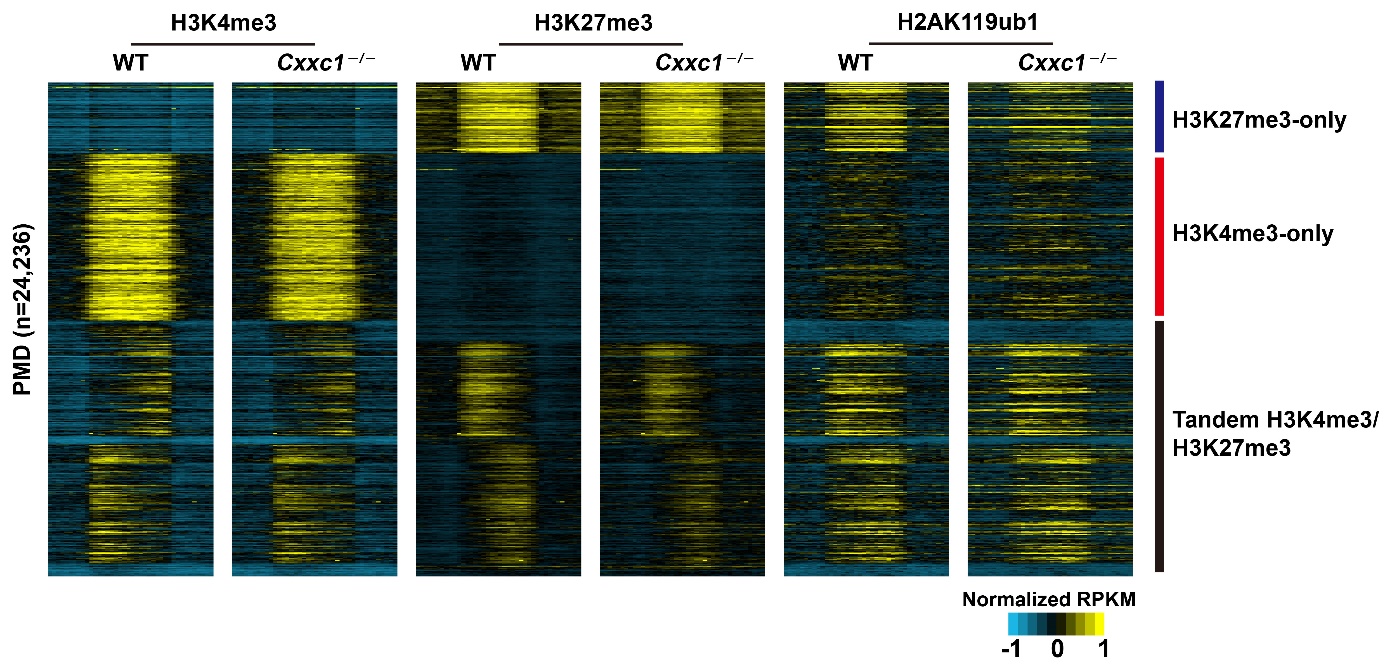


**Figure S2. Heat maps showing H3K4me3, H3K27me3, and H2AK119ub1 in partially-methylated domains in wild-type and *Cxxc1*-null oocytes.**

**Supplementary Tables**

**Table S1. Quality control of bisulfate sequencing of WT and Cxxc1-deleted GV oocytes.**

| **Sample** | **Total reads** | **Mapping efficiency** | **CpG methy ratio** | **CHG methy ratio** | **CHH methy ratio** | **CT conversion** |
| --- | --- | --- | --- | --- | --- | --- |
| WGBS-GV-WT1 | 25,380,121 | 36.1% | 38.985% | 3.987% | 3.663% | 0.993 |
| WGBS-GV-WT2 | 26,601,961 | 32.1% | 37.060% | 3.639% | 3.464% | 0.993 |
| WGBS-GV-WT3 | 28,760,810 | 33.2% | 36.347% | 3.581% | 3.430% | 0.993 |
| WGBS-GV-CZ1 | 30,924,841 | 33.3% | 31.417% | 1.648% | 1.716% | 0.993 |
| WGBS-GV-CZ2 | 27,105,235 | 33.1% | 29.077% | 1.692% | 1.806% | 0.993 |
| WGBS-GV-CZ3 | 26,636,341 | 33.1% | 33.965% | 1.789% | 1.867% | 0.993 |

**Table S2. Quality control of ChIP-seq data.**

| **Sample** | **Total reads** | **Mapping efficiency** | **Uniquely mapping efficiency** |
| --- | --- | --- | --- |
| WT-H3K4me3-rep1 | 13,004,340 | 98.01% | 69.96% |
| WT-H3K4me3-rep2 | 13,117,188 | 97.72% | 70.57% |
| WT-H3K27me3-rep1 | 28,726,087 | 92.06% | 56.18% |
| WT-H3K27me3-rep2 | 26,017,426 | 92.73% | 57.27% |
| WT-H3K36me3-rep1 | 18,009,570 | 61.07% | 49.57% |
| WT-H3K36me3-rep2 | 21,878,334 | 84.2% | 66.98% |
| WT-H2AK119ub1 | 25,174,747 | 80.63% | 61.2% |
| Cxxc1null-H3K4me3-rep1 | 15,577,747 | 97.49% | 66.42% |
| Cxxc1null-H3K4me3-rep2 | 22,410,920 | 95.89% | 67.65% |
| Cxxc1null -H3K27me3-rep1 | 26,763,835 | 95.9% | 62.55% |
| Cxxc1null -H3K27me3-rep2 | 18,481,511 | 65.42% | 45.45% |
| Cxxc1null -H3K36me3-rep1 | 18,024,654 | 47.97% | 39.37% |
| Cxxc1null -H3K36me3-rep2 | 18,070,758 | 68.1% | 55.4% |
| Cxxc1null -H2AK119ub1 | 29,358,577 | 70.56% | 56.84% |
